# Associations Between Single-Child Status and Metabolic Syndrome in Children and Adolescents in China

**DOI:** 10.3389/fped.2021.661164

**Published:** 2021-05-20

**Authors:** Manman Chen, Yanhui Li, Li Chen, Di Gao, Zhaogeng Yang, Ying Ma, Tao Ma, Bin Dong, Yanhui Dong, Jun Ma, Jie Hu

**Affiliations:** ^1^National Health Commission Key Laboratory of Reproductive Health, School of Public Health, Institute of Child and Adolescent Health, Peking University, Beijing, China; ^2^Menzies Health Institute Queensland, Griffith University, Brisbane, QLD, Australia

**Keywords:** one child policy, single children, metabolic syndrome, clustered CVD risk factors, adolescent

## Abstract

**Objective:** To evaluate the associations between single-child status and metabolic syndrome (MS) and to identify the highest risk group of MS among single children.

**Methods:** Differences in participants' characteristics by sex were examined by Student's *t*-test for continuous variables and Pearson's chi-squared test for categorical variables. Multivariate logistic regression analysis was performed to estimate the odds ratios (*OR*) and 95% confidence intervals (*CI*) for MS and its components according to the single-child status. Radar maps were used to compare the composition of different components in MS.

**Results:** In total, 11,784 (5,880 boys) children and adolescents were included in this study, with a mean age of (11.3 ± 3.1) years. MS was observed in 7.1% of participants, with a higher prevalence in boys (8.2%) than girls (5.9%) (*P* < 0.05). The prevalence of MS, elevated blood pressure and abdominal obesity in single children were higher than that in children with siblings, particularly in boys (*P* < 0.001). Elevated risk of abdominal obesity was observed in single children [boys (1.56, 95% CI: 1.31, 1.85), girls (1.40, 95% CI: 1.19, 1.63)], however, increased ORs of elevated blood pressure and metabolic syndrome were observed in single-child boys only (1.19, 95% CI: 1.01, 1.40 and 1.76, 95% CI: 1.34, 2.31, respectively). Results showed that a statistically significant association between single child status and MS was mainly observed in urban boys (2.04, 95% CI: 1.33, 3.12) and rural boys (1.50, 95% CI: 1.05, 2.15), but not in girls. Among all the combinations of MS, two combinations were significantly associated with the single-child status, including the combination of elevated blood pressure, abdominal and low HDL-C (1.45, 1.04, 2.04) and the combination of elevated blood pressure, abdominal obesity, low HDL-C and hypertriglyceridemia (2.04, 1.40, 3.06) (*P* < 0.05).

**Conclusions:** The present study found that single children and adolescents had a higher risk of MS, elevated blood pressure and abdominal obesity. The associations were stronger in urban boys. Further attention should be directed to the prevention and control strategies targeting the high-risk population of MS.

## Introduction

Metabolic syndrome (MS) (1), first coined by Haller and Hanefeld (2), is characterized as a cluster of five underlying cardiometabolic risk factors including dyslipidemia, abnormal glucose and insulin regulation, abdominal obesity and hypertension. It has been recognized that MS can lead to a series of adverse health consequences, including type 2 diabetes mellitus (T2DM) and cardiovascular disease (CVD) (3, 4). According to the estimation by Saklayen, MS was observed in 20–25% of adults globally (5). China has experienced a rapid increment of MS over the past decades from 2.4 to 15.5% (5, 6), and has become one of the highest risk areas.

MS occurred mostly in the adult population, especially in the elders (7). However, the MS development in children and adolescents is concerning as they may experience an incremental process of causes or precursors in their early life (8–11). Previous studies demonstrated that childhood metabolic disorders were significantly associated with MS in later life (12). Thus, it is important to prevent and manage MS during childhood in order to reduce the risk of cardiovascular disorders in later life.

The “one-child-per-couple” family planning policy (13), which aimed at easing the trend of population explosion. This policy has been launched in 1979 and has been enforced for more than 40 years in China. It was a basic state policy in China, which was one of the largest and most dramatic population-control campaigns in the world. Over the past decades, the policy worked well in containing the fast-growing population, which have significantly changed the family structure in China hosting the largest singleton population in the world (14, 15). In recent years, China released the two-child policy, yet the fertility rate was not simultaneously growing due to the ingrained only-child concepts. Meanwhile, we have also witnessed the economy booming and improved living standards in China, where the prevalence of cardiovascular and metabolic diseases has increased dramatically over the past decades (16).

In this context, attention has been raised about the health implications of single-child family structure. It has been reported that single children were associated with a higher risk of a series of adverse health consequences, such as obesity, impaired vision and mental disorders (17). However, limited research has assessed the association between single-child status and MS. As MS represents the outcome of evaluations to a cluster of biomarkers, which provides an alternative to the use of single parameters. The use of cluster biomarkers in identifying MS is less sensitive to measurement errors and compensates for day-to-day fluctuations observed in any single risk factor (18–20). Based on a large sample of Chinese children aged 7 to 18 years, the objectives of the present study were: (1) to evaluate the associations between the single-child status and MS, and its cardiometabolic risk components; (2) to identify the highest risk group of MS among single children.

## Methods

### Study Design and Population

This study used data from a cross-sectional survey of children and adolescents in seven provinces/cities of China, namely Hunan, Ningxia, Tianjin, Chongqing, Liaoning, Shanghai, Guangzhou. The study design has been reported in a previous study (21). Briefly, the study was designed as a multi-stage cluster sampling method involving 11784 children and adolescents in 2012. About 12 to 16 primary and secondary schools were randomly chosen from each province. Two classes in each grade were randomly selected in these schools. All students and their parents in these selected classes were invited to participate in the survey. Informed consents were signed before completing the survey questionnaire, physical measurement, and blood sample collection. All study sites followed the same protocol during the implementation process, and the randomization process was performed by a staff member, who was not involved in the survey. The project was approved by the Ethical Committee of Peking University (IRB No. 00001052-12072).

### Data Collection

All children who participated in this study undertook the anthropometric measurements and a venous blood sample collection, which were conducted by trained investigators. Besides, all parents were required to complete a structured self-administrated questionnaire at home.

### Anthropometric Measurements

Anthropometric measurements were conducted according to standard protocol and the measuring instruments were similar at all study sites. Children were required to remove their clothes and shoes and wear only their under wears for the measurements. Height was measured using the portable stadiometer with 0.1 cm precision, Waist circumference (WC) was measured with an accuracy of 0.1 cm using a non-elastic tape at the end of a natural breath at the midpoint between the top of the iliac crest and the lower margin of the last palpable rib, two measurements were recorded for each child and then was averaged as the final height and WC value. Blood pressure was measured using a mercury sphygmomanometer with the correct cuff size on the right arm of the participant in a relaxed, sitting position. Every participant was measured twice at 1-min intervals, the average of the two readings for systolic blood pressure (SBP) and diastolic blood pressure (DBP) were used for data analysis.

### Blood Sample Collection and Detection

A venous blood sample was collected in the morning after an overnight (at least 8 h) fasting. After 10 min seat rest, a blood sample was collected from the antecubital vein and then transfused into vacuum tubes. Blood specimens were transported in a chilled insulated container immediately after collected from venipuncture, and then frozen at −80°C after centrifuged at 2,000 g for 10 min. Plasma samples collected at each province/city were shipped by air in dry ice to the laboratory in Beijing, where the samples were stored at −80°C until laboratory detections were performed. Fasting lipid profile (in enzymatic methods), including high-density lipoprotein cholesterol (HDL-C) and triglyceride (TG), and fasting glucose (glucose oxidase meth) were tested by the automatic biochemistry analysis system.

### Questionnaire Survey

The children questionnaire was performed to collect basic information and lifestyle. The parental questionnaire was performed to collect information about singleton status, household income, parental BMI, family history of hypertension and diabetes. Singleton status information was obtained by asking “how many children do you have in your family?” If respondents answered that there was only one child in the family, their children were assigned to the “single-children” group, otherwise, children were assigned to the “not single-children” group. Household income was calculated as the sum of monthly income (in CNY) of all household members and classified as ≤ 5000, 5000–12000, or ≥12000 CNY. Parents were asked to report their height (cm) and weight (kg), and body mass index (BMI) was calculated as the weight (kg) divided by the square of the height (m^2^). BMI cut-offs of 24 and 28 were used to define parental overweight and obesity according to the criteria established by the Working Group on Obesity in China. To obtain the family history of hypertension and diabetes, we asked questions of “whether you have been diagnosed with hypertension by a doctor in a medical institution or have taken drugs to control blood pressure?” and “whether you have been diagnosed with diabetes mellitus by a doctor in a medical institution or taken drugs to control blood glucose?” We defined the child as having a family history if any parent responded “Yes.”

### MS Definition

According to the modified criteria of Adult Treatment Panel III (ATP III) defined by the National Cholesterol Education Program (NCEP) (22), MS refers to the presence of at least three of the following five components: (1) central obesity, WC > 90th percentile for respective sex and age; (2) elevated blood pressure, SBP or DBP > 90th percentile for respective sex, age, and height; (3) elevated TG, TG ≥ 1.24 mmol/L; (4) low HDL-C, HDL-C ≤ 1.03 mmol/L; and (5) elevated blood glucose, fasting glucose ≥5.6 mmol/L.

### Statistical Analysis

Mean and standard deviations (SD) were reported for continuous variables, and frequencies and percentages were reported for categorical variables. Differences in demographic and biochemical characteristics by sex were examined by Student's *t*-test for continuous variables and Pearson's chi-squared test for categorical variables. Differences in prevalence of MS and its components among sex and residence groups were tested using Pearson's chi-squared test. Multivariate logistic regression analyses were performed to estimate odds ratios (*OR*) and 95% confidence intervals (*CI*) for MS and its components according to single-child status. Potential confounders were adjusted in the multivariate logistic regression models, with age and residence adjusted in Model 1 and additional fruit, vegetables, physical activity, parental educational attainment, parental overweight, parental obesity, parental hypertension, and parental diabetes mellitus adjusted in Model 2. Radar maps were used to compare the different combinations of MS components to determine the major affected MS by the single-child status. All statistical analyses were conducted using SPSS 20.0 (IBM, NY, USA) and R software (Version 3.3.2, USA) for windows, and a two-sided *P* < 0.05 was considered statistically significant.

## Results

### Characteristics of the Participants

Demographic and biochemical characteristics of participants in single children and not-single children are presented in Table 1. In total, 11,784 (5,880 boys) children and adolescents were included in this study, with a mean age of 11.3 ± 3.1 years. The sample comprised 7,870 (4,159 boys) single children and 3,914 (1,730 boys) not-single children. Significant differences in sex, height, weight, BMI, WC, parental educational acquisition and parental overweight/obesity were observed between single children and not-single children (*P* < 0.001). The mean WC and fasting plasma glucose (FPG) level in single children were higher than those of not-single children (*P* < 0.001), but no statistically significant differences in SBP, DBP, HDL-C and TG level were observed between single children and not-single children.

**Table 1:** Demographic and biochemical characteristics of the participants between single children and not-single children.

**Variables**	**Total (*n* = 11,784)**	**Single (*n* = 7,870)**	**Not-single (*n* = 3,914)**	***p*-value**
Age, year	11.3 ± 3.1	11.3 ± 3.1	11.2 ± 3.0	0.093
**Sex**, ***n*** **(%)**				
Boys	5,880 (49.9)	4,159 (52.7)	1,730 (44.2)	**<0.001**
Girls	5,904 (50.1)	3,720 (47.3)	2,184 (55.8)	
**Residence area**, ***n*** **(%)**				
Urban	6,612 (56.1)	4,463 (56.7)	2,149 (54.9)	0.063
Rural	5,172 (43.9)	3,407 (43.3)	1,765 (45.1)	
Height, cm	148.6 ± 16.01	149.3 ± 16.2	147.0 ± 15.4	**<0.001**
Weight, kg	43.1 ± 15.7	44.0 ± 16.3	41.2 ± 14.2	**<0.001**
BMI	18.9 ± 3.9	19.1 ± 4.0	18.5 ± 3.6	**<0.001**
WC, cm	66.0 ± 10.9	66.6 ± 11.2	64.8 ± 10.1	**<0.001**
SBP, mmHg	104.7 ± 12.0	104.7 ± 12.1	104.5 ± 11.7	0.417
DBP, mmHg	66.2 ± 8.9	66.2 ± 8.9	66.3 ± 8.9	0.413
HDL-C, mg/dL	52.8 ± 12.6	52.9 ± 12.5	52.6 ± 12.9	0.214
TG, mg/dL	81.8 ± 41.1	82.0 ± 42.2	81.5 ± 38.6	0.524
FPG, mmol/L	4.7 ± 0.6	4.8 ± 0.6	4.7 ± 0.7	**<0.001**
**Parental educational acquisition**				
Middle school or below	4,298 (36.6)	2,055 (26.2)	2,243 (57.6)	**<0.001**
High school	3,289 (28.0)	2,239 (28.6)	1,050 (27.0)	
Junior college	1,972 (16.8)	1,634 (20.8)	338 (8.7)	
College or above	2,172 (18.5)	1,911 (24.4)	261 (6.7)	
**Parental OW or OB**, ***n*** **(%)**				
None	6,415 (54.4)	4,393 (55.8)	2,022 (51.7)	**<0.001**
Either or both	5,369 (45.6)	3,477 (44.2)	1,892 (48.3)	
**Parental hypertension**				
Yes	840 (7.1)	542 (6.9)	298 (7.6)	0.120
No	10,510 (89.2)	7,057 (89.7)	3,453 (88.2)	
Missing/Refusal	434(3.7)	271 (3.4)	163 (4.2)	
**Parental diabetes**				
Yes	259 (2.2)	179, (2.3)	80 (2.0)	0.482
No	10,639 (90.3)	7,132 (90.6)	3,507 (89.6)	
Missing/Refusal	886 (7.5)	559 (7.1)	327 (8.4)	

*Mean ± SD or n(%); OW, Overweight; OB, Obesity. Bold values represent P < 0.05*.

### Prevalence of MS in Single and Not-Single Children

Table 2 summarized the prevalence of MS and its components stratified by sex. In total, about 7.1% of participants were observed with MS with a higher prevalence in boys (8.2%) than girls (5.9%) (*P* < 0.05). Furthermore, the prevalence of elevated blood pressure, abdominal obesity, low HDL-C, hypertriglyceridemia and impaired fasting glucose were 21.9, 23.1, 13.8, 16.9, and 3.4%, respectively. The prevalence of MS, elevated blood pressure and abdominal obesity in single children were higher than that in not-single children among children and adolescents, particularly in boys (*P* < 0.001).

**Table 2:** A comparison of the prevalence of CVD risk factors between single children and not-single children, stratified by sex.

**Variable**	**Total**	**Single**	**Not-single**	*p*-value^*^
**Total**					
Elevated BP	2,577 (21.9)	1,765 (22.4)	812 (20.7)	**0.038**
Abdominal obesity	2,720 (23.1)	1,979 (25.1)	741 (18.9)	**<0.001**
Low HDL-C	1,628 (13.8)	1,058 (13.4)	570 (14.6)	0.097
Hypertriglyceridemia	1,990 (16.9)	1,339 (17.0)	651 (16.6)	0.603
Impaired fasting glucose	397 (3.4)	285 (3.6)	112 (2.9)	**0.031**
Metabolic syndrome	832 (7.1)	613(7.8)	219 (5.6)	**<0.001**
**Boys**					
Elevated BP	1,490 (25.3)	1,101 (26.5)	389 (22.5)	**0.001**
Abdominal obesity	1,326 (22.6)	1,030 (24.8)	296 (17.1)	**<0.001**
Low HDL-C	889 (15.1)	629 (15.2)	260 (15.0)	0.901
Hypertriglyceridemia	868 (14.8)	641 (15.4)	227 (13.1)	**0.022**
Impaired fasting glucose	271 (4.6)	202(4.9)	69 (4.0)	0.143
Metabolic syndrome	485 (8.2)	390 (9.4)	95 (5.5)	**<0.001**
**Girls**					
Elevated BP	1,087 (18.4)	664 (17.8)	423 (19.4)	0.146
Abdominal obesity	1,394 (23.6)	949 (25.5)	445 (20.4)	**<0.001**
Low HDL-C	739 (12.5)	429 (11.5)	310 (14.2)	**0.003**
Hypertriglyceridemia	1,122 (19.0)	698 (18.8)	424 (19.4)	0.539
Impaired fasting glucose	126 (2.1)	83 (2.2)	43 (2.0)	0.501
Metabolic syndrome	347 (5.9)	223 (6.0)	124 (5.7)	0.617

*n (%)*;

* *Pearson's chi-squared tests. Bold values represent P < 0.05*.

### Association of Single-Child Status and MS

Results of logistic regressions were presented in Tables 3, 4. We found that the single-child status was associated with a higher prevalence of elevated blood pressure, abdominal obesity, and metabolic syndrome. In terms of gender difference, an elevated risk of abdominal obesity was observed in single children [boys (1.56, 95% CI: 1.31, 1.85), girls (1.40, 95% CI: 1.19, 1.63)], but increased ORs of elevated blood pressure and MS were observed in single-child boys only [elevated blood pressure (1.19, 95% CI: 1.01, 1.40), metabolic syndrome (1.76, 95% CI: 1.34, 2.31)].

**Table 3:** Multivariate odds ratios (OR) and 95% confidence intervals (CI) of MS according to single child status, stratified by sex.

**Metabolic syndrome parameter**	**Total**		**Boys**		**Girls**	
**Elevated BP**						
Model 1	1.09 (0.99, 1.20)	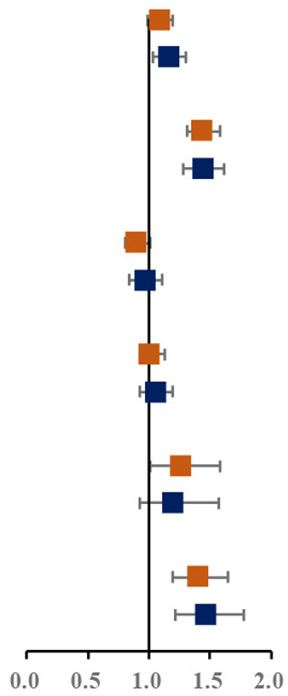	**1.16 (1.01, 1.34)**	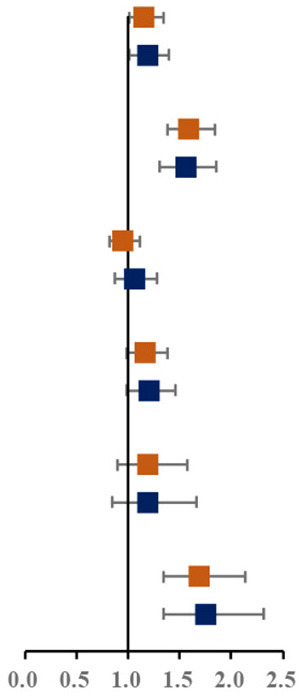	0.92 (0.80, 1.05)	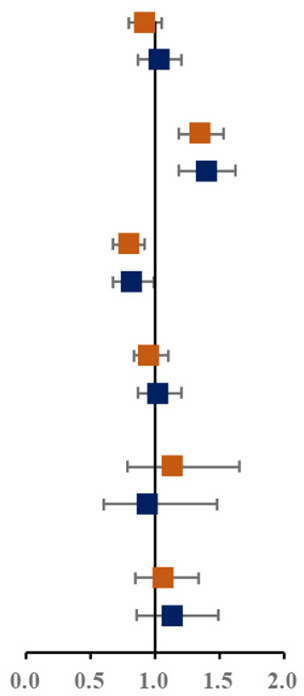
Model 2	**1.16 (1.03, 1.30)**		**1.19 (1.01, 1.40)**		1.03 (0.87, 1.21)	
**Abdominal obesity**						
Model 1	**1.44 (1.31, 1.58)**		**1.60 (1.38, 1.84)**		**1.35 (1.19, 1.53)**	
Model 2	**1.44 (1.28, 1.62)**		**1.56 (1.31, 1.85)**		**1.40 (1.19, 1.63)**	
**Low HDL-c**						
Model 1	0.90 (0.81, 1.01)		0.96 (0.82, 1.12)		**0.80 (0.67, 0.92)**	
Model 2	0.97 (0.84, 1.11)		1.06 (0.87, 1.28)		**0.82 (0.67, 0.99)**	
**Hypertriglyceridemia**						
Model 1	1.01(0.92, 1.13)		1.17(0.99, 1.38)		0.96(0.84, 1.10)	
Model 2	1.05(0.93, 1.19)		1.20(0.99, 1.46)		1.02(0.87, 1.21)	
**High fasting glucose**						
Model 1	**1.27(1.01, 1.58)**		1.20(0.90, 1.58)		1.14(0.79, 1.66)	
Model 2	1.20 (0.92, 1.57)		1.19 (0.85, 1.66)		0.94 (0.60, 1.48)	
**Metabolic syndrome**						
Model 1	**1.41 (1.20, 1.65)**		**1.69 (1.34, 2.14)**		1.07 (0.85, 1.34)	
Model 2	**1.47 (1.22, 1.78)**		**1.76 (1.34, 2.31)**		1.13 (0.86, 1.49)	

*Model 1: adjusted for age, residence area*.

*Model 2: adjusted for age, residence area, physical activity, fruits, vegetables, parental educational attainment, parental overweight/obesity, parental hypertension, parental diabetes mellitus. Bold values represent *P* < 0.05*.

**Table 4:** Multivariate odds ratios (OR) and 95% confidence intervals (CI) of metabolic syndrome according to single child status, stratified by residence-sex.

**Metabolic syndrome parameter**	**Urban boys**		**Urban girls**		**Rural boys**		**Rural girls**	
Elevated BP								
Model 1	**1.47 (1.20, 1.80)**	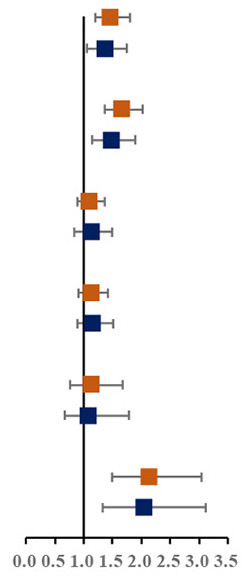	0.99 (0.81, 1.21)	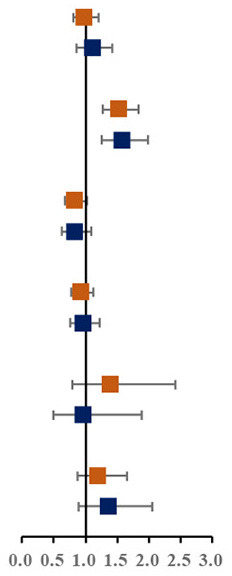	0.92 (0.76, 1.12)	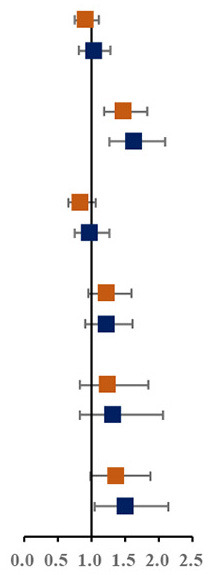	0.86 (0.71, 1.04)	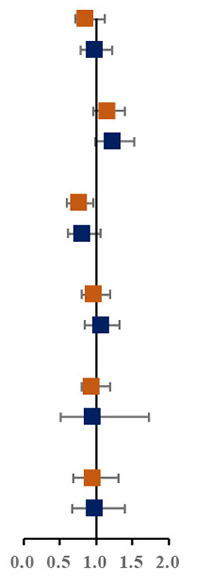
Model 2	**1.36 (1.05, 1.75)**		1.11 (0.86, 1.42)		1.03 (0.82, 1.29)		0.98 (0.79, 1.22)	
Abdominal obesity								
Model 1	**1.67 (1.37, 2.03)**		**1.53 (1.28, 1.83)**		**1.48 (1.20, 1.83)**		1.16 (0.96, 1.39)	
Model 2	**1.47 (1.15, 1.89)**		**1.58 (1.25, 1.98)**		**1.63 (1.27, 2.09)**		1.23 (0.99,1.53)	
Low HDL-c								
Model 1	1.10 (0.88, 1.37)		0.83 (0.67, 1.02)		0.84 (0.67, 1.06)		0.76 (0.60, 0.96)	
Model 2	1.12 (0.84, 1.49)		0.83 (0.63, 1.09)		0.97 (0.75, 1.27)		0.81 (0.62, 1.07)	
Hypertriglyceridemia								
Model 1	1.13 (0.91, 1.41)		0.94 (0.78, 1.13)		1.23 (0.96, 1.60)		0.97 (0.80, 1.19)	
Model 2	1.15 (0.88, 1.51)		0.96 (0.76, 1.22)		1.22 (0.91, 1.62)		1.06 (0.84, 1.33)	
High fasting glucose								
Model 1	1.13 (0.76, 1.68)		1.39 (0.80, 2.41)		1.25 (0.84, 1.85)		0.94 (0.57, 1.58)	
Model 2	1.08 (0.66, 1.79)		0.96 (0.49, 1.89)		1.31 (0.83, 2.06)		0.95 (0.52, 1.73)	
Metabolic syndrome								
Model 1	**2.14 (1.50, 3.04)**		1.20 (0.87, 1.66)		1.37 (0.99, 1.88)		0.95 (0.69, 1.31)	
Model 2	**2.04 (1.33, 3.12)**		1.36 (0.90, 2.06)		**1.50 (1.05, 2.15)**		0.97 (0.68, 1.40)	

*Model 1: adjusted for age, residence area*.

*Model 2: adjusted for age, residence area, physical activity, fruits, vegetables, parental educational attainment, parental overweight/obesity, parental hypertension, parental diabetes mellitus. Bold values represent P < 0.05*.

We further assessed the associations among sex-residence stratified groups (Table 4). Results showed that a statistically significant association between single-child status and MS was mainly observed in urban boys (2.04, 95% CI: 1.33, 3.12) and rural boys (1.50, 95% CI: 1.05, 2.15), but not in girls.

### Combination of Different Components of MS Among Single and Not-Single Children

**Figure 1:**
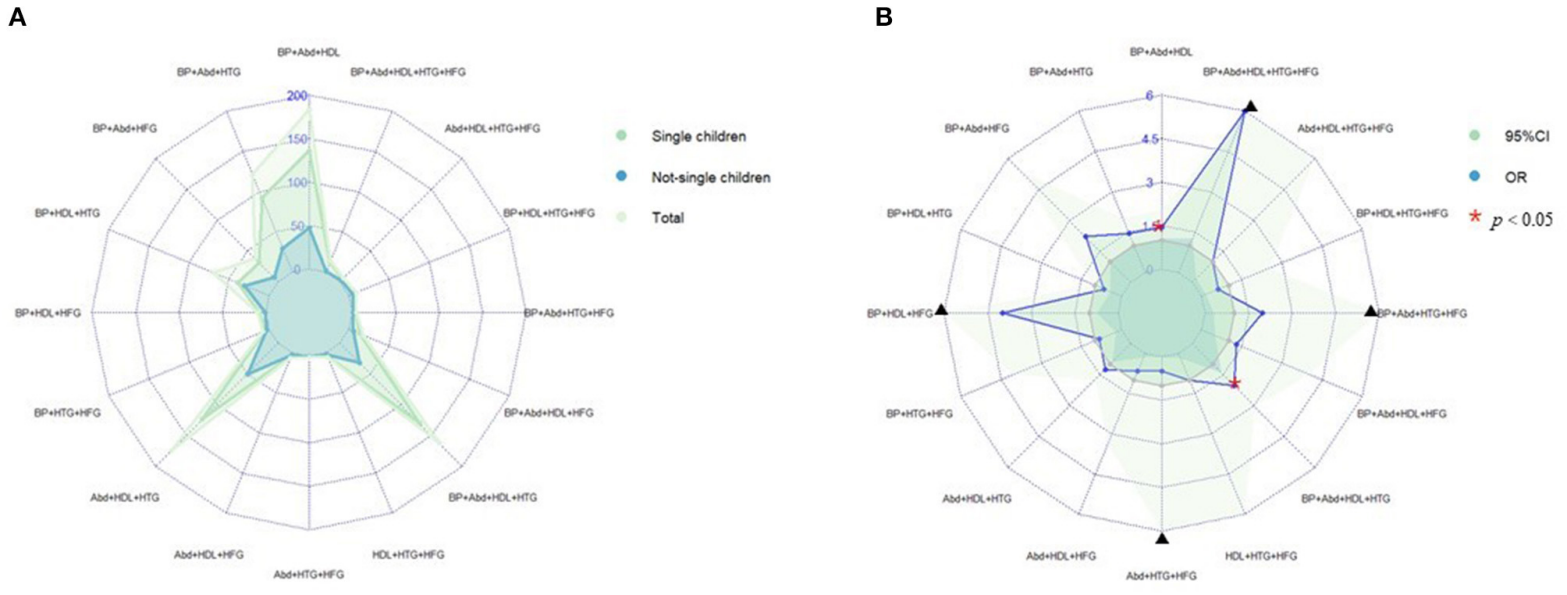
**(A)** Prevalence of various components of MS among single and not-single children. **(B)** Risk of various components of MS among single and not-single children. BP, Elevated blood pressure; Abd, Abdominal obesity; HDL, Low HDL-C; HTG, Hypertriglyceridemia; HFG, High fasting glucose. ^▴^Represents 95% confidence interval is over 6.

Prevalence of various components of MS among single and not-single children is presented in Figure 1A. Among the sixteen combinations of MS, the combination of elevated blood pressure, abdominal obesity and low HDL-C, the combination of abdominal obesity, low HDL-C and hypertriglyceridemia, and the combination of elevated blood pressure, abdominal obesity, low HDL-C and hypertriglyceridemia were the top three highest prevalent MS combinations (1.6, 1.5, and 1.4%, respectively). The proportion of three combinations of MS in single children were higher than that in not-single children. The associations between different MS combinations and single-child status were shown in Figure 1B. Among all the combinations of MS, there were two combinations with higher prevalence and ORs, including the combination of elevated blood pressure, abdominal and low HDL-C (1.45, 95% CI: 1.04, 2.04) and the combination of elevated blood pressure, abdominal obesity, low HDL-C and hypertriglyceridemia (2.04, 95% CI: 1.40,3.06).

## Discussion

Based on a national representative epidemiological survey among Chinese children and adolescents, the present study found that single children could have a higher risk of MS and its components including elevated blood pressure and abdominal obesity. However, no association was observed between single-child status and risk of MS components of low HDL-C, hypertriglyceridemia or high fasting glucose. In addition, there were significant associations between single-child status and risk of MS in urban and rural boys, but not in girls. Further analyses revealed that the combination of elevated blood pressure, abdominal obesity and low HDL-C accounted for the highest proportion of MS among single children.

Using the ATP III criteria, we found a prevalence of metabolic syndrome (7.1%) in this study, which was higher than the result from Chinese Nutritional and Health Surveillance conducted in 2010–2012 (4.2%). The prevalence was also likely to be higher than what was found by the investigation conducted by the Chinese Work Group of Pediatric Metabolic Syndrome (1.4% using MS-IDF2007 criteria and 2.4% using MS-CHN2012 criteria) (6). Similar prevalence has been observed in Korean adolescents (6.7% in total, 8.5% in boys and 4.5% in girls using ATP III criteria) (23) and higher prevalence was reported in studies from Italian (24) and Mexican (25) children. A study in Brazilian reported a lower prevalence (3.6%) than that in our study (26). Consequently, children and adolescents in China face a high risk of MS.

Compared to children with siblings, single children had a significantly higher risk of obesity (27). One-child policy had linked to the child-obesity epidemic in China, which could be attributable to over-feeding and not sharing with siblings (28). Previous studies raised concerns regarding the health implications of single children, such as imbalanced sex ratio (29), impaired vision, and mostly, obesity (17, 30). Additionally, it was concerning single children were indulged by parents and grandparents, forming their unhealthy personalities (28). In this study, we found that single children were associated with a higher risk of abdominal obesity in both boys and girls, which was consistent with previous studies (31). The associations between single-child status and abdominal obesity in boys and girls were comparable in this study, whereas Li et al. suggested the association in girls was weaker than that in boys (32). Nevertheless, the findings of this study provided evidence of the high MS risk in single children, which could be associated with resource allocation, family coddling and the tolerance of bad behavioral factors.

Previous studies about the relationship between single-child status and adverse health consequences mainly focused on obesity, with insufficient investigations on other metabolic risks. To our knowledge, this was the first study in China assessing the associations between single-child status and the risk of childhood MS and its components. Our study suggested that single-child status has a stronger influence on pediatric MS in boys than in girls, especially among urban boys. Higher-income, high fat or protein diet, and the growing consumption of high-energy snacks could be the potential reasons leading to the elevated risk of pediatric MS in these high-risk population (33). A possible mechanism is that a high-energy diet will cause insulin resistance, producing excessive energy accumulation, which the body cannot dissolve through normal metabolism (34, 35). Previous studies also found that boys spend a lot of time playing games and watching TV, and thus have less time for physical exercise, which increases the risk of metabolic diseases (36–38). Last but not least, this study identified that the combination of elevated blood pressure, abdominal obesity and low HDL-C as the highest prevalent metabolic abnormalities in single children, suggesting that future efforts in controlling high blood pressure, BMI, and blood lipid level should be prioritized in public health propositions and actions (39, 40).

## Limitation

This study encompasses large number of participants, allowing stratified analysis of correlations by sex and residence. However, there are some limitations. First, the data was from a cross-sectional survey, which limited our ability to demonstrate causal inferences about the relationship between one-child status and pediatric metabolic syndrome. Further research based on longitudinal data is needed to confirm the causal inference about the relationship. Second, potential confounding factors including children's lifestyle, family socioeconomic status, and other relating factors were not included in the analysis Further studies should assess the impact of these possible confounding factors. Third, there may be recall bias in data collected through the self-administered questionnaire.

## Conclusions

The present study found that single children and adolescents had a higher risk of MS and its components including elevated blood pressure and abdominal obesity. However, no significant associations were observed between single-child status and risk of other MS components of low HDL-C, Hypertriglyceridemia and high fasting glucose. In addition, the associations were stronger in urban boys than those in urban girls, rural boys, and rural girls. What's more, among the sixteen combinations of MS, the combination of elevated blood pressure, abdominal obesity and low HDL-C was most prevalent in single children and adolescents. This study relates to the growing number of single children induced by the one-child policy and the rising prevalence of MS, providing novel insights into an important health consequence of one-child policy in China. For the metabolic health status of single children, further attention should be directed to the prevention and control strategies targeting abdominal obesity and elevated blood pressure and other high-risk population.

## Data Availability Statement

The datasets presented in this article are not readily available because the dataset apply to children and adolescents. Requests to access the datasets should be directed to Manman Chen, 1911210173@pku.edu.cn.

## Ethics Statement

The studies involving human participants were reviewed and approved by Medical Ethical Committee of Peking University Health Science Center (IRB No. 00001052-12072). Written informed consent to participate in this study was provided by the participants' legal guardian/next of kin.

## Author Contributions

MC and YL performed the data analysis. MC, YL, LC, DG, ZY, YM, and TM interpreted results, wrote, and finalized the manuscript. BD, YD, JM, and JH reviewed and revised the manuscript. All authors contributed to conception, design of this study, read, and approved the final manuscript.

## Conflict of Interest

The authors declare that the research was conducted in the absence of any commercial or financial relationships that could be construed as a potential conflict of interest.
